# Timing and Spatial Distribution of Loess in Xinjiang, NW China

**DOI:** 10.1371/journal.pone.0125492

**Published:** 2015-05-13

**Authors:** Yun Li, Yougui Song, Libin Yan, Tao Chen, Zhisheng An

**Affiliations:** 1 State Key Laboratory of Loess and Quaternary Geology, Institute of Earth Environment, Chinese Academy of Sciences, Xi’an, 710061, China; 2 Joint Center for Global Change Studies, Beijing 100875, China; Institute of Botany, CHINA

## Abstract

Central Asia is one of the most significant loess regions on Earth, with an important role in understanding Quaternary climate and environmental change. However, in contrast to the widely investigated loess deposits in the Chinese Loess Plateau, the Central Asian loess–paleosol sequences are still insufficiently known and poorly understood. Through field investigation and review of the previous literature, the authors have investigated the distribution, thickness and age of the Xinjiang loess, and analyzed factors that control these parameters in the Xinjiang in northwest China, Central Asia. The loess sediments cover river terraces, low uplands, the margins of deserts and the slopes of the Tianshan Mountains and Kunlun Mountains and are also present in the Ili Basin. The thickness of the Xinjiang loess deposits varies from several meters to 670 m. The variation trend of the sand fraction (>63 μm) grain-size contour can indicate the local major wind directions, so we conclude that the NW and NE winds are the main wind directions in the North and South Xinjiang, and the westerly wind mainly transport dust into the Ili basin. We consider persistent drying, adequate regional wind energy and well-developed river terraces to be the main factors controlling the distribution, thickness and formation age of the Xinjiang loess. The well-outcropped loess sections have mainly developed since the middle Pleistocene in Xinjiang, reflecting the appearance of the persistent drying and the present air circulation system. However, the oldest loess deposits are as old as the beginning of the Pliocene in the Tarim Basin, which suggests that earlier aridification occurred in the Tarim Basin rather than in the Ili Basin and the Junggar Basin.

## Introduction

The Chinese Loess Plateau (CLP), with its continuously deposited loess-paleosol sequences, records the thickest, largest, oldest and most continuous aeolian deposits on Earth. Together with the marine sediments and polar ice cores, these deposits constitute one of the three most commonly used proxies in paleoclimate studies. Over the past two decades, this unique continental archive has been extensively studied with the goal to understand the history and cause of Asian aridification, dust transportation, East Asian circulation and Tibetan Plateau uplift [1–12].

Xinjiang is one of the most significant loess regions in China and is located between the extensive East Europe loess to the west and the well-studied CLP loess to the east. It is a crucial area for clarifying the interactions between the Asian monsoon and the westerlies. Obruchev [13] proposed that loess in the northwest Junggar basin is aeolian silt transported via northwesterly winds. Liu [14] holds a similar opinion but argued that the dust was transported by the monsoon from Mongolia. Recent research has shown that the Xinjiang loess was mainly derived from the adjacent deserts, including the Gurbantunggut Desert, Taklimakan Desert and Sary-Ishikotrau Desert [15–19].

Although the loess deposits in the Ili, Tarim and Jungger basins have been studied with respect to their ages, pedostratigraphy, rock magnetism, particle size, provenance, and elemental composition [9, 15–29], many discrepancies still exist. The dating reliability [22, 30–35] and the paleoclimatic significance of the proxies [9, 21, 22, 33, 36] are hotly debated. The chronology of Central Asian loess has remained a long-term unsolved problem, especially for luminescence and radiocarbon dating. Some studies suggest that optically stimulated luminescence (OSL) ages are in good agreement with the observed stratigraphy in the field [22]. However, most OSL and ^14^C ages are believed to be underestimated, possibly due to pedoturbation during pedogenesis [34], sample contamination [22], or anomalous fading [32, 35]. Magnetic susceptibility has long been used as a proxy of pedogenesis intensity and East Asian summer monsoon strength in the CLP [37, 38]. However, the magnetic properties of the Xinjiang loess may be dominated by wind intensity and source mineralogy, rather than pedogenesis [18, 27, 39]. Unlike the CLP, there is no obvious differentiation in most elements of the Xinjiang loess-paleosol sequences, and the long-term variable trend of elements are therefore not ideal proxies to study the weathering history in Xinjiang [29, 40]. Moreover, the heat-moisture pattern is still controversial. Tree ring records have shown that the climate change pattern is warm-dry and cold-wet in eastern Kazakhstan [41], whereas it is warm-wet and cold-dry in eastern Xinjiang [42].

Based on field investigations and topsoil grain-size analyses, in addition to summarizing previous literature, this paper will perform the following three objectives: (1) investigate the distribution and thickness of loess in Xinjiang, (2) compare the pedostratigraphy and age of the Xinjiang loess, and (3) discuss the factors that control the distribution, thickness and age of the Xinjiang loess.

## Physical settings

**Fig 1:**
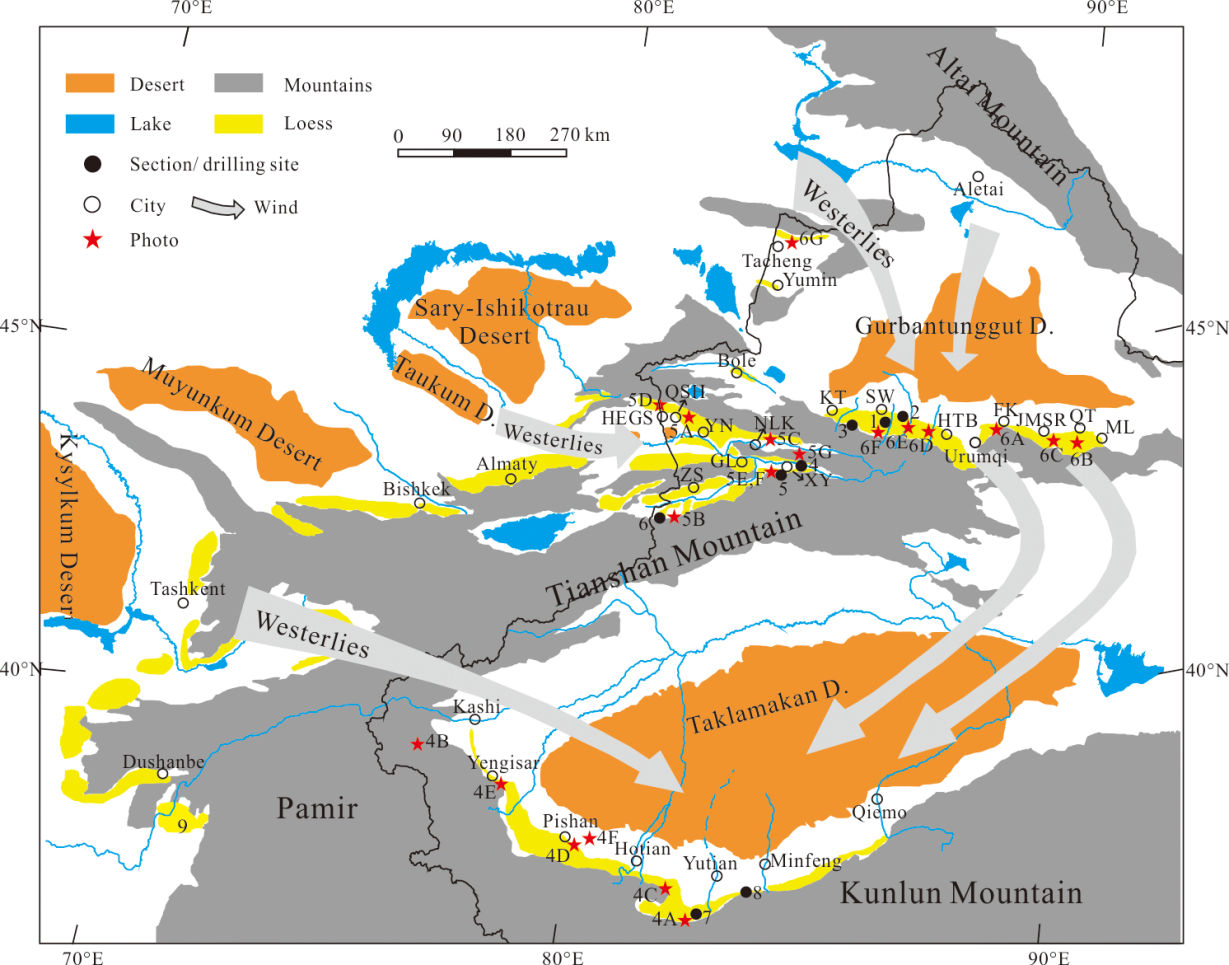
The distribution of loess and the locations of the investigated loess sections in Xinjiang. The pictures of loess drillings and sections (4A-4F, 5A-5G, and 6A-6G) are shown in Figs 4, 5, and 6, and the strata of the numbered sections (1–9) are shown in Fig 7. ML: Mulei, QT: Qitai, JMSE: Jimsar, FK: Fukang, HTB: Hutubi, SW: Shawa, KT: Kuitun, NLK: Nilke, XY: Xinyuan, ZS: Zhaosu, GL: Gongliu, YN: Yining, QSH: Qingshuihe, HEGS: Huoerguosi.

Xinjiang (75°-90°E, 35°-45°N) is located at the center of the Eurasian continent and covers over 1.6 million km^2^. From north to south, Xinjiang is composed of the Altay Mountains, Junggar Basin, Tianshan (including the North Tianshan, South Tianshan mountains and the Ili Basin), Tarim Basin and Kunlun Mountains (Fig 1). The Taklimakan Desert (330,000 km^2^) and Gurbantunggut Desert (48,800 km^2^), the first and second largest deserts in China, are located at the center of the Tarim Basin and Junggar Basin, respectively.

**Fig 2:**
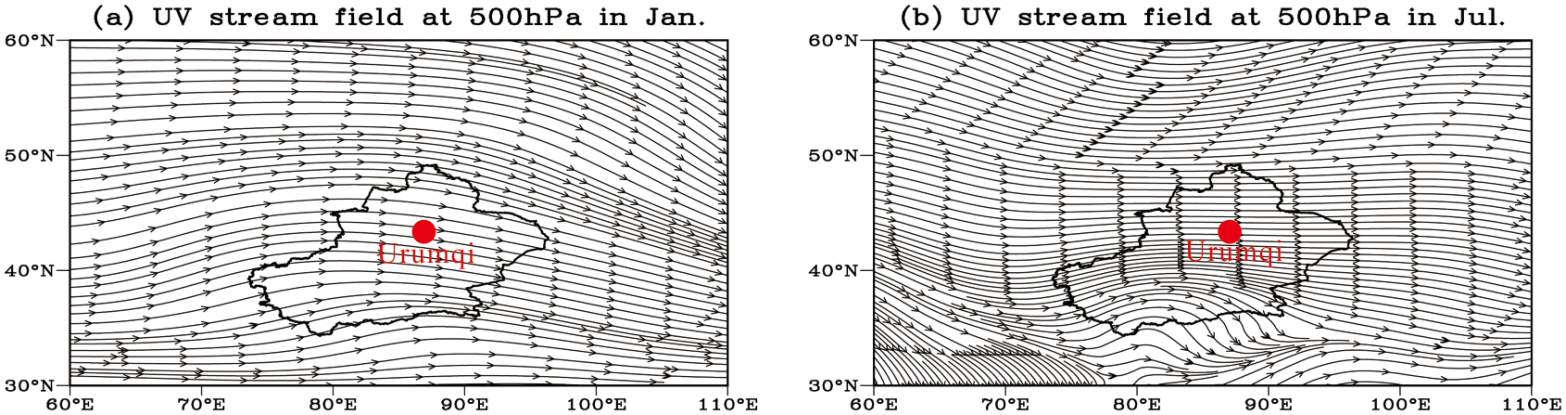
The UV stream field at 500 hPa (approximately 5500 m a.s.l.) in Xinjiang: (a) January and (b) July. Red circle denotes the Urumqi, and the black outline shows the outline of the Xinjiang province. Red circle denotes the Urumqi, and the black outline shows the outline of the Xinjiang province.

**Fig 3:**
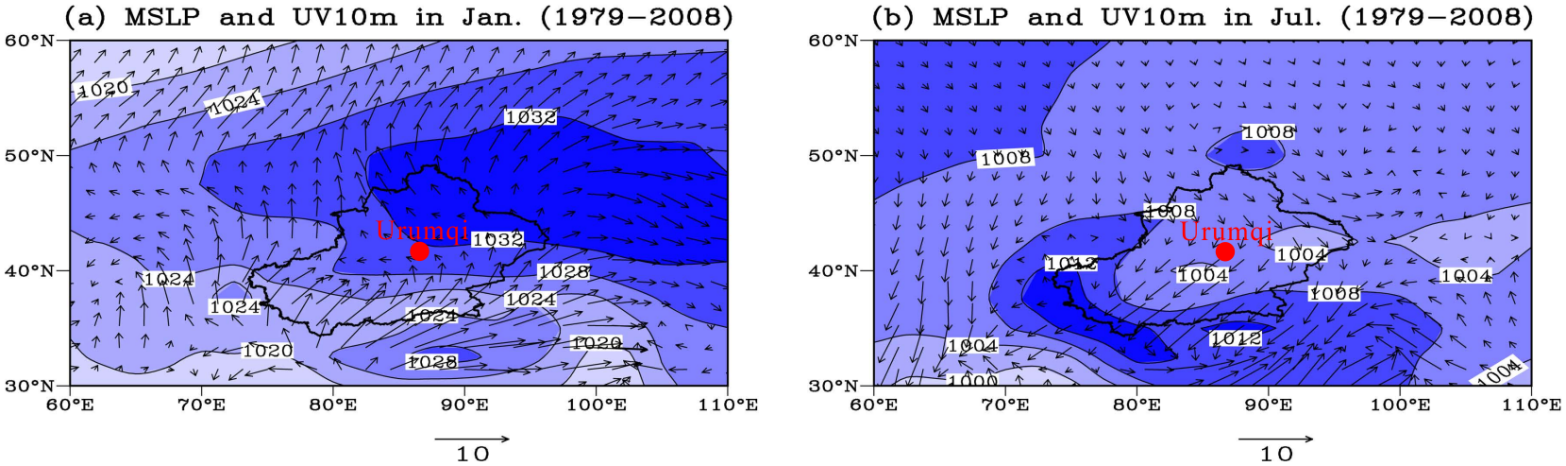
The atmospheric pressure field at sea level (the blue shaded region, hPa) and the UV 10 m wind circulation (m/s) in Xinjiang: (a) January and (b) July. Red circle denotes the Urumqi, and the black outline shows the outline of the Xinjiang province.

Xinjiang is an arid and semi-arid region, climatologically dominated by mid-latitude westerlies, Siberian High-Pressure systems and the Indian Monsoon [43, 44]. These different wind systems are dominant in different regions of Xinjiang, and each area has a distinct average annual temperature and precipitation as a function of altitude and topography. The Ili Basin, an intermontane basin defined by west-facing trumpet-shaped mountains, is climatically controlled by the westerlies (Fig 2), which carry adequate moisture from the Atlantic Ocean, Mediterranean Sea, Black Sea and Caspian Sea [45]. The mean annual precipitation in the basin is the highest in Xinjiang and is 200–400 mm on the plains but can reach 800 mm in the mountain zones [46]. The mean annual temperature in the Ili basin varies from 2.6 to 9.2°C depending on the terrain. Northern Xinjiang (the Junggar Basin) features an arid temperate continental climate controlled by mid-latitude westerlies and Siberian High Pressure, especially during the winter [47]. The mean annual air temperature in this region is approximately 5°C, and the mean annual precipitation ranges from 60–150 mm in the desert to 150–400 mm in the surrounding mountainous areas (Fig 3); however, the potential evapotranspiration is approximately 1000–3500 mm/year. The climate of Southern Xinjiang (the Tarim Basin), which is influenced by the Siberian high-pressure cells, mid-latitude westerlies and the Indian Monsoon [43], is characterized by extreme aridity. The northeast winds occupy southern Xinjiang, not only at the surface but also in the upper atmosphere, especially during the summer (Figs 2 and 3). The mean annual precipitation of southern Xinjiang is rather low, ranging between 30 to 200 mm, whereas its mean annual evaporation reaches 2536 mm. Its mean temperature is no less than -10°C in January and reaches 26.5°C in August [47].

Semi-desert and steppe vegetation dominates the Tianshan and Kunlun Mountains. The vegetation in Xinjiang exhibits obvious vertical differentiation. From higher to lower elevations, there is the alpine cushion-like vegetation zone, the alpine meadow zone, the subalpine meadow zone, the montane forest-meadow zone, the montane steppe and the desert zone [48].

## Samples and Methods

During recent years, we performed a series of investigations of the aeolian sands, loess deposits and landforms in Xinjiang. Fieldwork has included investigations of the loess distribution and thickness measurements of the loess. To avoid human disturbance, samples were collected in natural vegetation-covered areas with no signs of erosion, cultivation or burning and that are as distant as possible from farmland, villages, and towns. A total of 189 topsoil samples (2–5 cm depth) were collected in the study area (75°-90°E, 35°-45°N), including 101 samples from the northern Tianshan Mountains, 50 samples from the Ili basin, and 38 samples from the Tarim Basin. Any leaves and/or roots were removed in the field. These samples represent two belts (sand desert and grassland soil) and exclude Gobi gravels, alluvial and fluvial sediments. All samples were collected on private land; therefore, please contact Li Haomin for future permissions. Our sampling locations did not require specific permissions and did not involve endangered or protected species.

For the grain size analysis, all samples were pretreated by adding 30% hydrogen peroxide (H_2_O_2_) and 6 N hydrochloric acid (HCL) to remove organic matter and calcium carbonate. The remains were dispersed with a 0.5 N sodium metaphosphotate ((NaPO_3_)_6_) solution and ultrasonicated for 10 min before measuring. Samples were measured using a Malvern Mastersizer 2000 laser grain-size analyzer at the Institute of Earth Environment, Chinese Academy of Sciences. This instrument has a measurement range of 0.01–2000 μm with a 0.1Φ interval resolution.

## Results

### Spatial distribution

The distribution of the Xinjiang loess, so-called ‘piedmont loess’, is largely controlled by topography and wind direction. Loess deposits can be preserved not only on river and debris fan terraces, but also on piedmont slopes of high mountain ranges. The main distribution areas include the northern slopes of the Tianshan and Kunlun Mountains, as well as the Ili Basin [19].

**Fig 4:**
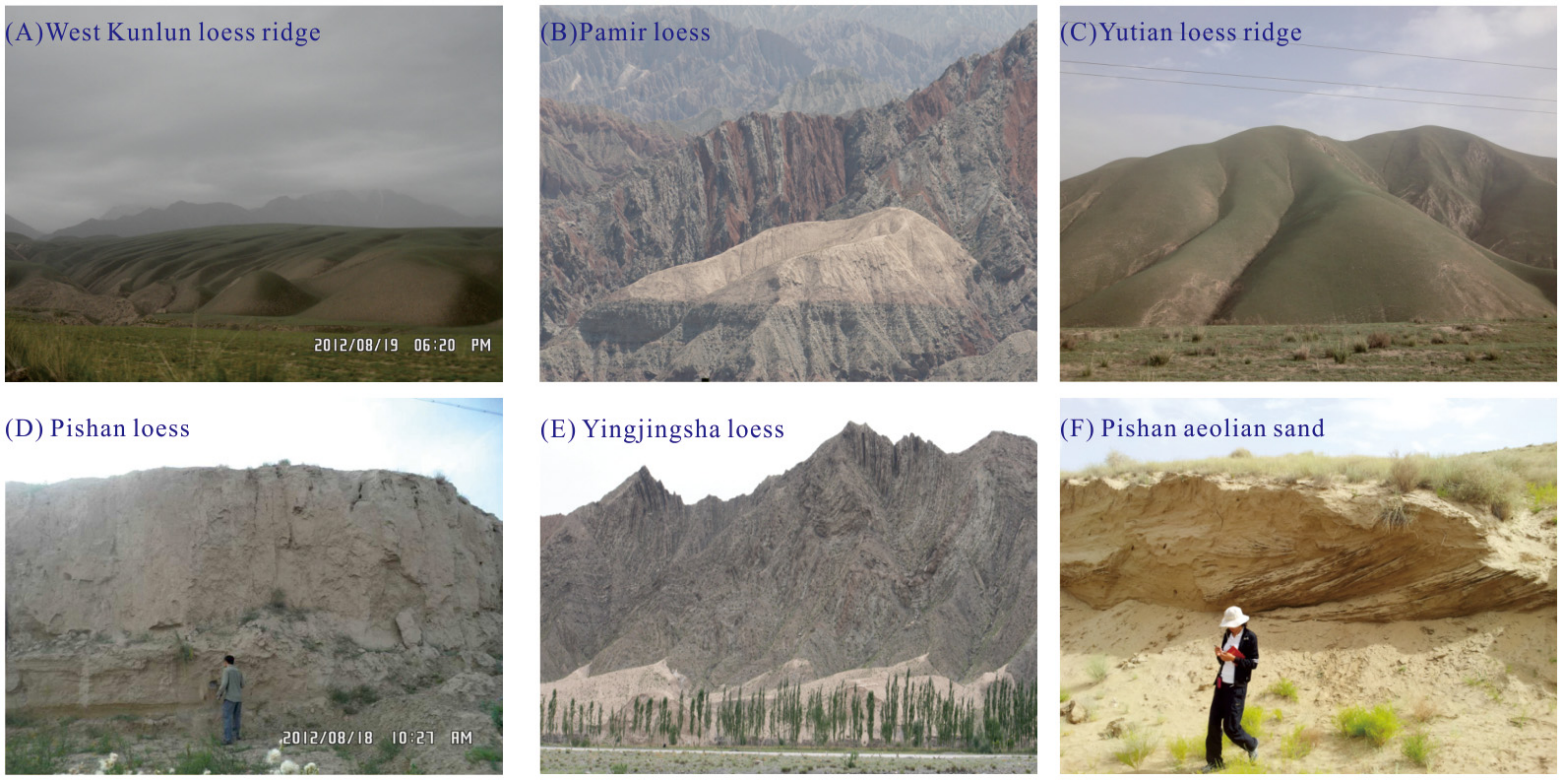
Photographs of loess at the northern slope of the Kunlun Mountains.

Loess is widely spread from Kashi in the west to Qiemo in the east on the northern slope of the Kunlun Mountains, with an elevation ranging from 2000 m to 4500 m a.s.l. (Fig 1). Unlike the so-called ‘Loess Yuan’ (the broad, flat, high tablelands) in the CLP, the Kunlun loess is characterized by loess ridges (Fig 4A). Loess in the Kunlun Mountains is not restricted by geomorphologic factors and covers strata of different ages. From piedmonts to high mountains, the loess exhibits a gradual decrease of thickness and content of fine materials [26]. On the Pamir Plateau to the west of the Kunlun Mountains, thin fragmental loessic rocks can be found on bedrock below 4000 m a.s.l. (Fig 4B). The loess thickness on river terraces in the Hotian-Yutian area is generally between 60 and 80 m in thickness (Fig 4A and 4C); however, on the broad flat watershed of the Keriya River, drilling core data indicates that the thickness can reach up into 670 m [27]. Above 3400 m a.s.l., the loess abruptly becomes thin, and small loess patches approximately 1 m thick may occur even at elevations above 5000 m a.s.l. [49–51]. Loess in the Kashi and Pishan area is only 8–10 m thick (Fig 4D and 4E). Between Minfeng and Qiemo, there are small thinning-southeastward loess mantles that are less than 2–3 m thick [19].

**Fig 5:**
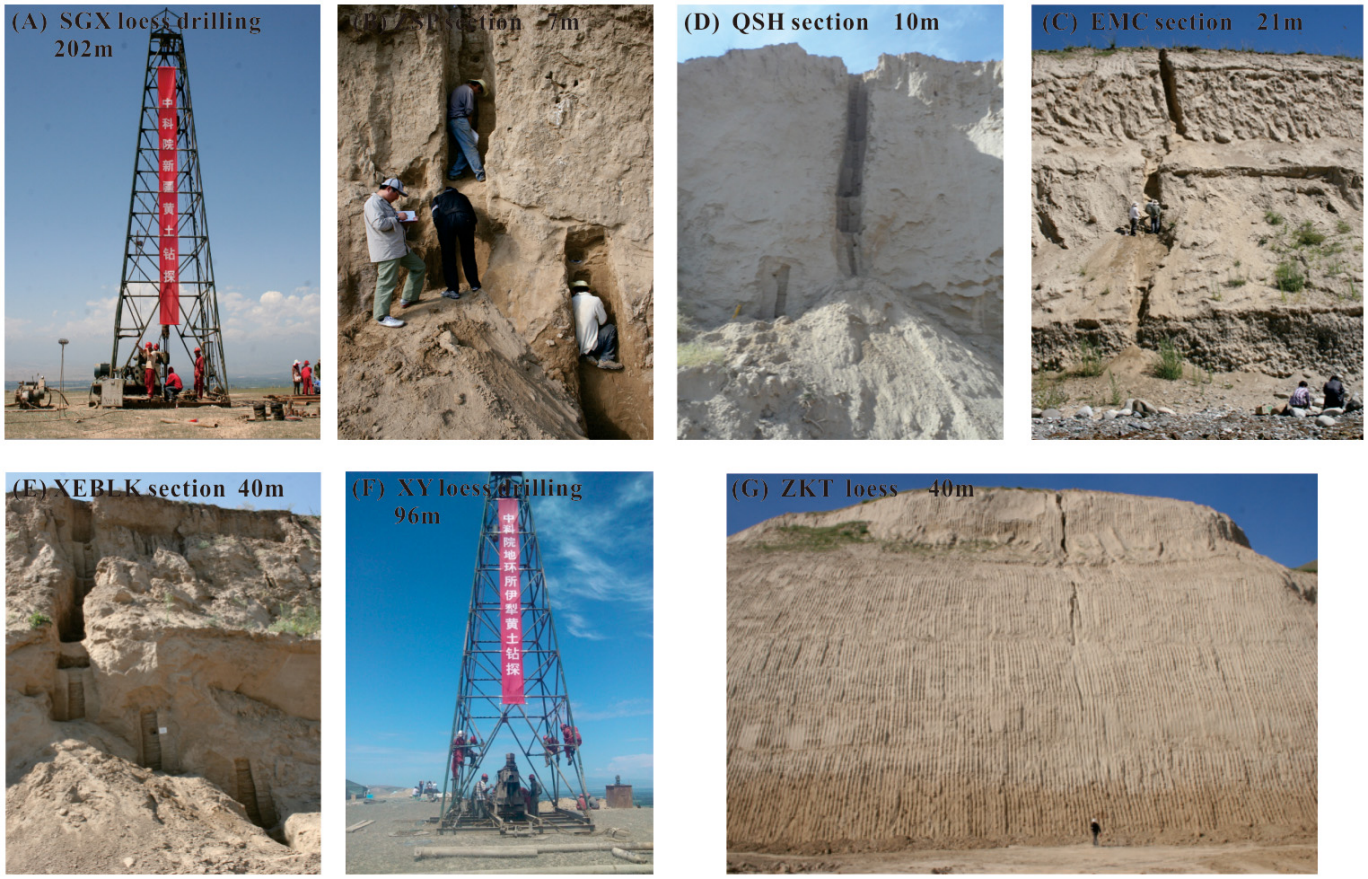
Photographs of loess sections and cores in the Ili Basin. SGX: Sangongxiang loess Drilling in Huocheng County; ZSP: Zhaosu Poma section in the south of the Ili Basin; QSH: Qingshuihe section in the Huoerguosi Economic Development Zone; EMC: Ermuchang loess in Nilke County.

In the Ili Basin, loess is widely distributed on river terraces from the southern slope of the North Tianshan Mountains to the northern slope of the South Tianshan Mountains. Previous research has indicated that from west to east along the Ili Basin, the loess forms a lens shape; in other words, it thins from the middle (Xinyuan County) towards both the east and west [17]. However, a recent Xinjiang loess-focused scientific drilling project revealed that the loess on the highest terrace of the Ili River around Qingshuihe is approximately 202 m thick (Fig 5A) [17] and is by far the thickest reported loess sediment in the Ili Basin. In the Zhaosu basin, loess that is several to tens of meters thick (Fig 5B) covers the Tekes River terraces and the foothills of the Tianshan Mountains, with elevations ranging from 1300 m to 2100 m a.s.l. [17]. Between 1250 and 1700 m, on the second terrace of the Kashi River, the loess sediment is more than 20 m thick (Fig 5C). The thickness of the loess is less than 20 m in Yining City (Fig 5D) and Gongliu County, with elevations ranging between 600 and 1600 m a.s.l. Around Xinyuan County, the loess between 900 and 1500 m a.s.l. is more than 80 m thick (Fig 5E) and reaches up to approximately 96 m in the Xinyuan drilling core (Fig 5F) but decreases to 40–50 m at Zeketai (Fig 5G). Around Nalati (1400 m a.s.l.), the loess is only several meters thick and gradually decreases and finally disappears toward the headwaters of the Kunes River.

**Fig 6:**
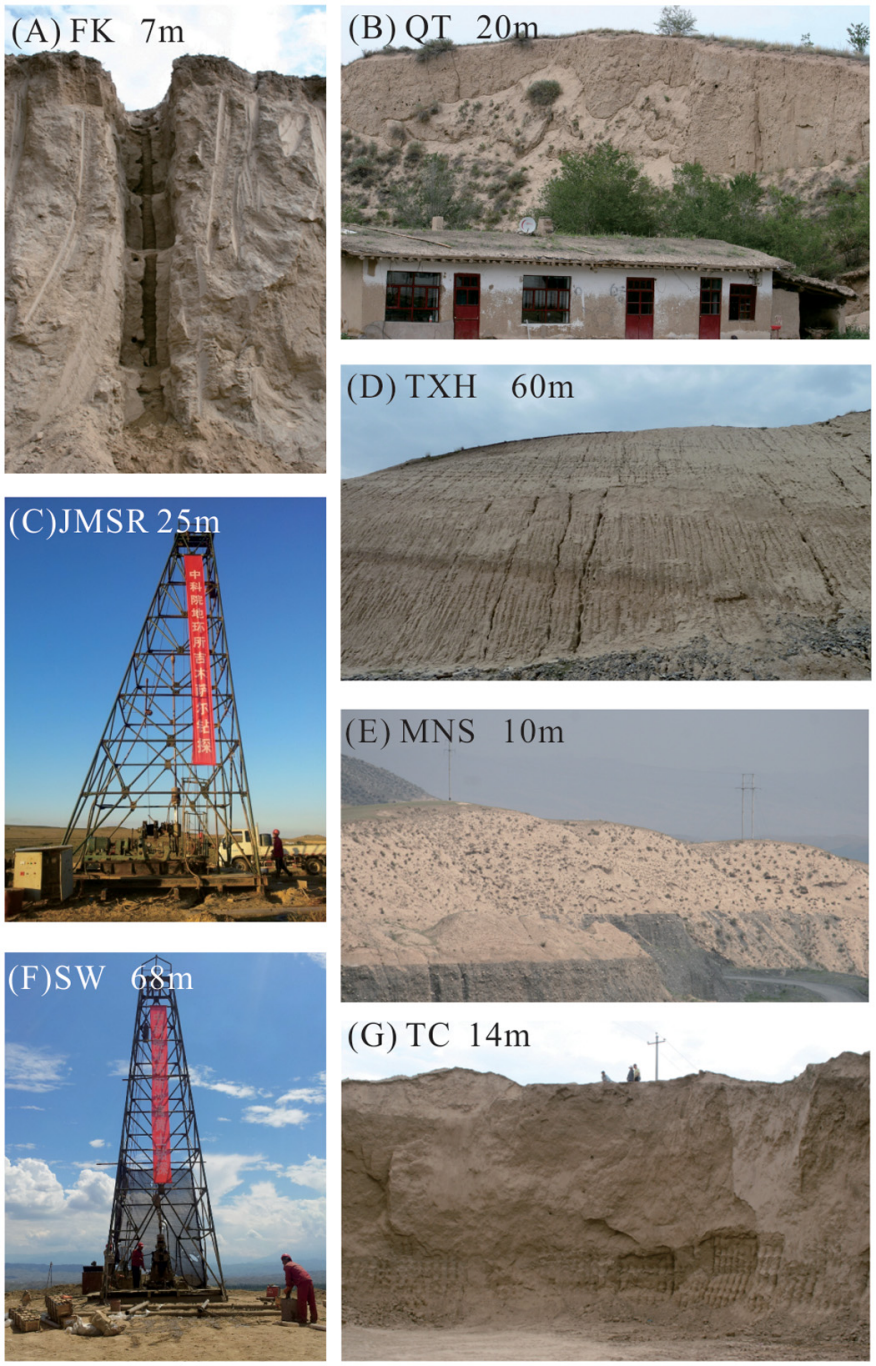
Photographs of loess sections and drillings from the northern slope of the Tianshan Mountains. FK: Fukang loess section in Fukang City; QT: Qitai loess section in Qitai County; JMSR: Jimsar loess drilling in Jimsar County; TXH: Taxihe loess on the north slope of the Tianshan Mountains; MNS: Manas loess on the terrace of the Manas River; SW: Shawan loess drilling in Shawan County; TC: Tacheng loess section in Tacheng City in the west of the Junggar Basin.

Along the southern margin of the Junggar Basin and below the fir-spruce forest on the northern slope of the Tianshan Mountains, loess forms a WNW-ESE belt (Fig 1) [16, 19], which is distributed on different geomorphic units (e.g., river terraces and foothills) between 700 and 2400 m a.s.l. With increasing elevation, the loess exhibits lentoid-distributed features, and the loess thickens away from the Junggar Basin to the south and pinches out further south adjacent the Tianshan Mountains [16]. Although there are extensive loess deposits to the north of the Tianshan Mountains, their distribution is not continuous, and the thickness varies in different places. In general, the loess between Fukang and Mulei is less than 20 m thick (Fig 6A, 6B and 6C) and thinner than 40 m at Urumqi. To the west of Urumqi, the widespread loess on the piedmont (Fig 6D) [52] and the Manas River terrace (Fig 6E) between Kuitun and Hutubi is between 5–30 m, but it can be as thick as between 30–50 m. These deposits even reach up to ~81 m thick on the second terrace of the Ningjia river [16] in Shawan (Fig 6F), and this is the thickest loess deposit on the north of the Tianshan Mountains. To the west of Kuitun, the loess becomes rapidly thinner and even disappears, whereas the loess around Bole gradually thickens from west to east and is approximately 2–20 meters thick on the terrace of the Bortala River. The thickness of the loess sediments ranges from several meters to 30 m between Tacheng and Yumin in the western Junggar Basin (Fig 6G). The maximum elevation of loess deposits to the north of the Tianshan Mountains is approximately 2400 m a.s.l., with the majority of elevations falling between 1000–1400 m a.s.l.

### Stratigraphic correlation and age

Due to the different dominant climatic systems, regional topography and aeolian sources, the Xingjiang loess is apparently different from those of the CLP. First, the Xinjiang loess units are generally pale yellow or grayish yellow, with little stratification, and are homogenous and massive with occasionally outcropping pellicle and fleck calcium carbonate. The paleosols are pale brown or grayish brown and may contain numerous terrestrial snail fossils and ferruginous mottles. However, most of the paleosol and loess units are difficult to distinguish because of the poor pedogenesis [18, 25, 53]. Second, paleoclimatic proxies, such as geochemical elements [17, 54], rock magnetism [27], mineral composition and quart surface texture [46], reveal that the Xinjiang loess experienced a much drier climate, weaker chemical weathering and poorer pedogenesis than the loess deposits in the CLP. Finally, the Xinjiang loess is described as a ‘silty loess’ due to its proximity to the source areas; the loess consists mainly of medium to coarse silt, which is in contrast to the finer-grained loess of the CLP [55].

Many magnetostratigraphy and absolute dating techniques (OSL, ^14^C and electron spin resonance (ESR)) have been conducted on the loess-paleosol sequences in Xinjiang during the past few years [15, 16, 22, 27, 30, 33, 56]. Although the ages of the Xinjiang loess remain controversial (as discussed above), we tentatively try to establish a regional pedostratigraphy by compiling published work to help reveal the regional paleoclimate and paleoenvironment of Xinjiang.

There are only a few papers that focus on the ages of the loess in the Kunlun Mountains. In the early 21st century, Fang et al. [15, 16] noted that loess deposits on the northern Kunlun Mountains were formed at approximately ~0.88 Ma, based on paleomagnetic dating. Recently, Zan [53] assigned an age of 0.95 Ma for the upper 207 m loess deposit in this region and suggested that the whole 671 m of loess was deposited since 3–4 Ma. Much works suggested that the oldest loess deposited since the beginning of the Pliocene [58–63]. In addition to the thick loess sections/drilling cores mentioned above, many thin sections have been found deposited on various geomorphic surfaces. Tang et al. [57] reported a 4.92 m thick loess section on the third terrace of the Keriya River in the northern Kunlun Mountains, and the dating of this deposit suggests that it spans more than the last 5,000 years. The ^14^C and luminescence dating results suggested that the Kunlun Mountains loess formed primarily after the last glaciation [58–63], which is equivalent to the age of the Malan loess in the CLP.

**Fig 7:**
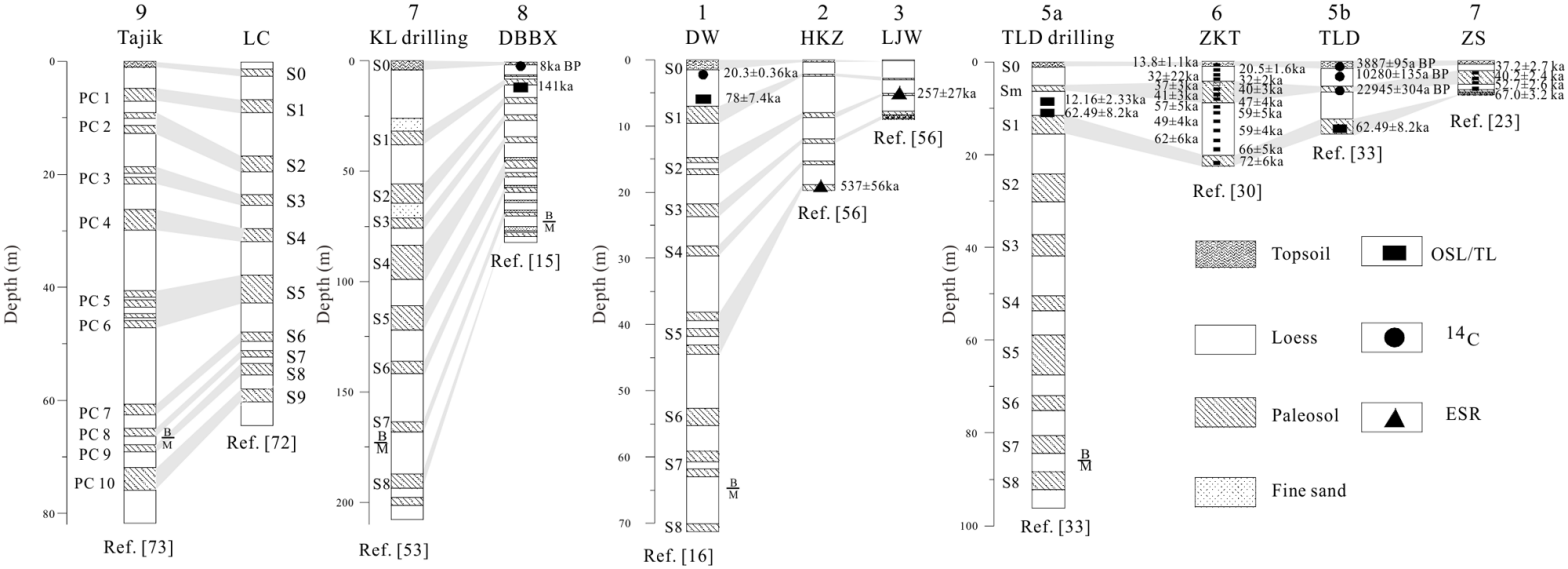
Stratigraphic correlations of outcropping loess sections and loess drillings in Xinjiang. Data sources: Tajik from Ref.73; LC from Ref.72; KL drilling from Ref. 28; DBBX from Ref.15; DW from Ref.16; HKZ and LJW from Ref.76; TLD drilling from Ref.34; ZKT from Ref.31; TLD from Ref.34; ZS from Ref.23.

In the Ili Basin, the oldest loess was dated to nearly 860 ka by paleomagnetic dating (Fig 7) [33]. However, most of the Ili loess strata formed after the last interglacial, which is equal to the age of the Malan loess in the CLP (Fig 7). Fig 7 shows that an Sm weak paleosol layer (equal to MIS 3) at a higher elevation (e.g., Zhaosu County) is thicker than that at a lower elevation (e.g., Talede Town). Because precipitation increases with increasing elevation, this suggests that more precipitation leads to stronger pedogenesis [20] and a thicker paleosol. For a more detailed description of the distributions and ages of the Ili loess, please refer to reference [17].

On the northern slopes of the Tianshan Mountains, several sections have been studied [39, 40, 56]. However, these studies focus on rock magnetism and geochemistry analysis to study the regional paleoenvironment rather than dating the loess-paleosol sequences. The bottom of the 81 m thick Dongwan loess-paleosol sequence on the highest terrace of the Ningjia River was estimated to be ~0.8 Ma by paleomagnetic dating result (Fig 7) [16]. However, Lu et al. [56] noted that the second paleosol of the 8 m thick Lujiaowa loess-paleosol sequence on the same terrace of the Manas River was dated to be 257 ka and inferred that the lowest paleosol belonged to S3 (~280–320 ka). They also suggested that this terrace was abandoned at ~0.3 Ma. Because Lujiaowan section is located at the foot of the Tianshan Mountain, the denudation maybe erode the bottom of the Lujiaowan section, so the age of Lujiaowan section is younger than that of the Dongwan loess-paleosol sequence. Based on ESR dating, the Hankazi section, approximately 20 m thick, was suggested to have formed since ~537 ka, which is equivalent to paleosol S5a in the CLP [56]. Moreover, a mid-Pleistocene mammalian fossil was found in the loess section, providing further support for a middle Pleistocene age for the oldest loess on the north of the Tianshan Mountains [64].

### The sand fraction of >63 μm grain size contours

**Fig 8:**
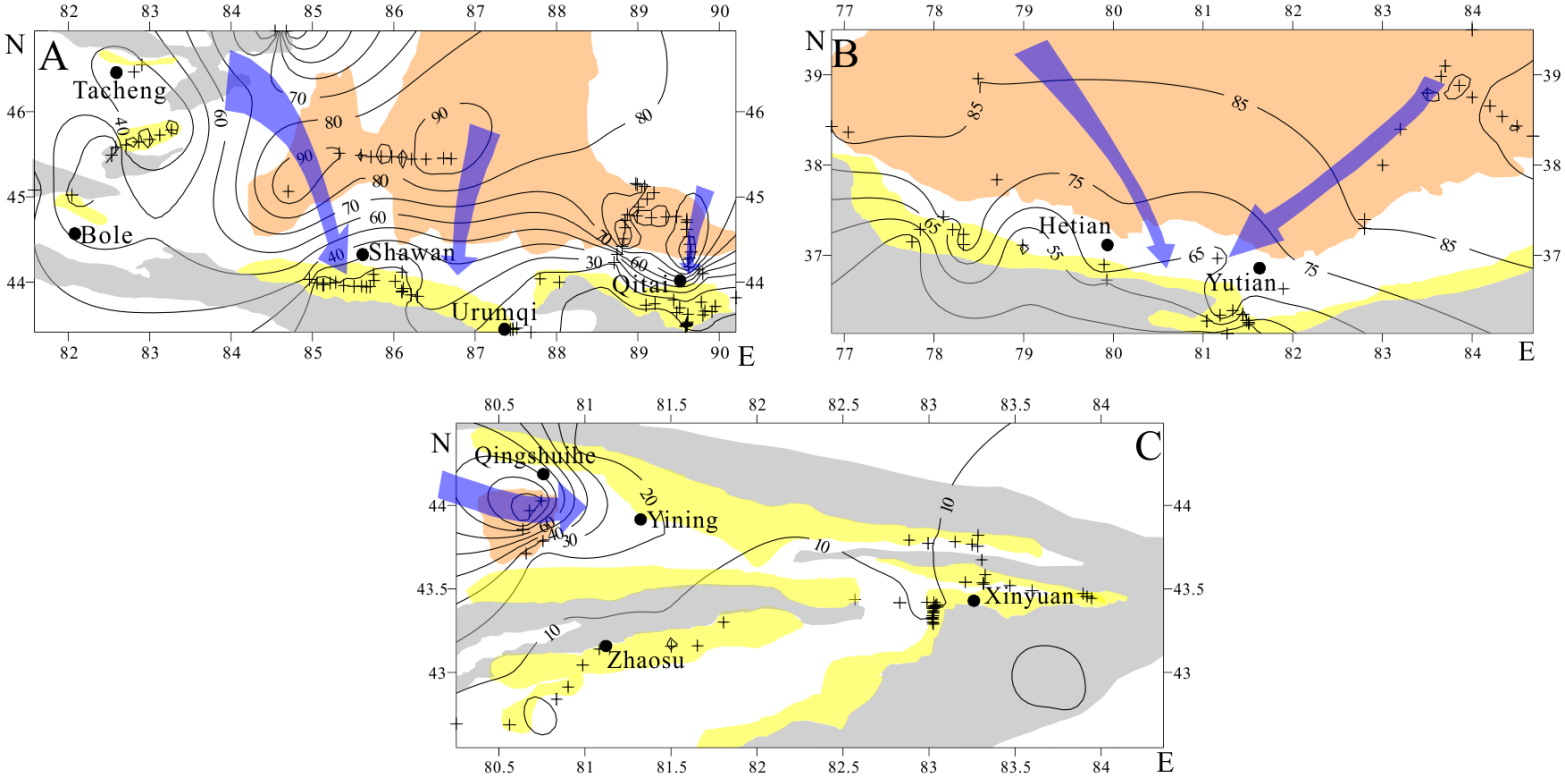
Map showing the contours of the >63 μm particle component of topsoil in Xinjiang: (A) the Junggar Basin; (B) the Tarim Basin; and (C) the Ili Basin. Cross symbols represent our sampling sites, dots represent the city/county, and arrows represent the possible wind direction.

Grain-size contour maps of the >63 μm sand fraction of the Xinjiang topsoil are shown in Fig 8. Due to various distances to source regions, the grain size of the Xinjiang surface soils differ from each other. Latitudinally, from desert to loess, the >63 μm (Fig 8A and 8B) particle content of topsoil ranges from 90% to 20% between Shihezi and Qitai in the Junggar Basin and 85% to 40% between Hotian and Yutian in the Tarim Basin. The grain-size contours become increasingly dense (i.e., >63 μm grain-size component changes more rapidly) and are concentrated on the northern slope of the Tianshan Mountains and Kunlun Mountains because the wind speed decreases when the wind is forced to pass over high mountains. This phenomenon further suggests that the Gurbantunggut Desert and the Taklimakan Desert are the main sources of loess for the northern Tianshan and Kunlun Mountains, respectively [19]. For the north of the Gurbantunggut Desert, the contours of the >63 μm particle content (Fig 8A) present a negative trend with increasing distance away from the Gurbantunggut Desert, with the content ranging from 90% to 30%. This pattern is in accord with the different regional landscape zones, including desert, shrub, and grassland, along with increasing altitude [54]. The >63 μm grain-size contours of surface sand of the Junggar Basin are more dense than those of the Tarim Basin (Fig 8A and 8B), especially around the loess-desert interaction region on the piedmont of high mountains (Fig 1). The topsoil particles >63 μm in diameter in the loess zone only account for 20% in the Tianshan Mountains, whereas the proportion reaches up to 50% in the Kunlun Mountains. Scarce precipitation and strong evaporation lead to the extreme aridity of the Tarim Basin, and stronger wind can transport coarser dust to deposits in the downwind zones on the northern slopes of the Kunlun Mountains. Meteorological observation data have also demonstrated that sandstorms occur more frequently in South Xinjiang than in North Xinjiang due to their different climates [65]. Fig 8A depicts the spatial variations of the >63 μm particle content around Tacheng in the western Junggar Basin. In general, it displays an evident closed-curve (Fig 8B) and mainly ranges between 30% and 50%, which decreases from the outer zones to the interior. This pattern indicates that decreasing wind speed leads to the increased settling of fine grains and an apparent intermontane basin defined by the local topography (Fig 1). The contours of >63 μm particle content component (Fig 8C), from west to east in the Ili Basin, change from dense to sparse with values shifting from 70% to 10%, especially on the western part of the Ili Basin. However, Fig 8C illustrates that in the east and southwest Ili basin around Zhaosu, the >63 μm grain-size content only accounts for 10% or less. This phenomenon is correlated with the varying distance from the source region, reduced wind speed caused by increasing elevation, and the blocking effect of the Tianshan Mountains.

## Discussions

### Significances of the coarse grain-size contours

Studies by Vandenberghe et al. [66] and Porter [67] demonstrated that three Chinese Loess Plateau particle-size zones are consistent with a proximal northwest source and transportation by the Asian winter monsoon, which results in deposition of a systematic northwest-southeast, time-transgressive spatial pattern demonstrated by median grain size. This pattern further revealed that during dust transportation, wind sorting is an important factor in the abrasion and size reduction of the dust particles [68]. Moreover, the >63 μm component (i.e., sand-sized grains) is conveyed in saltation or modified saltation mode near the ground surface [55]. Therefore, we further inferred that the perpendicular direction of the concentrated >63 μm grain-size contours represents the dust transportation direction, which is the regional wind direction.

### Formation mechanisms of the Xinjiang loess

**Fig 9:**
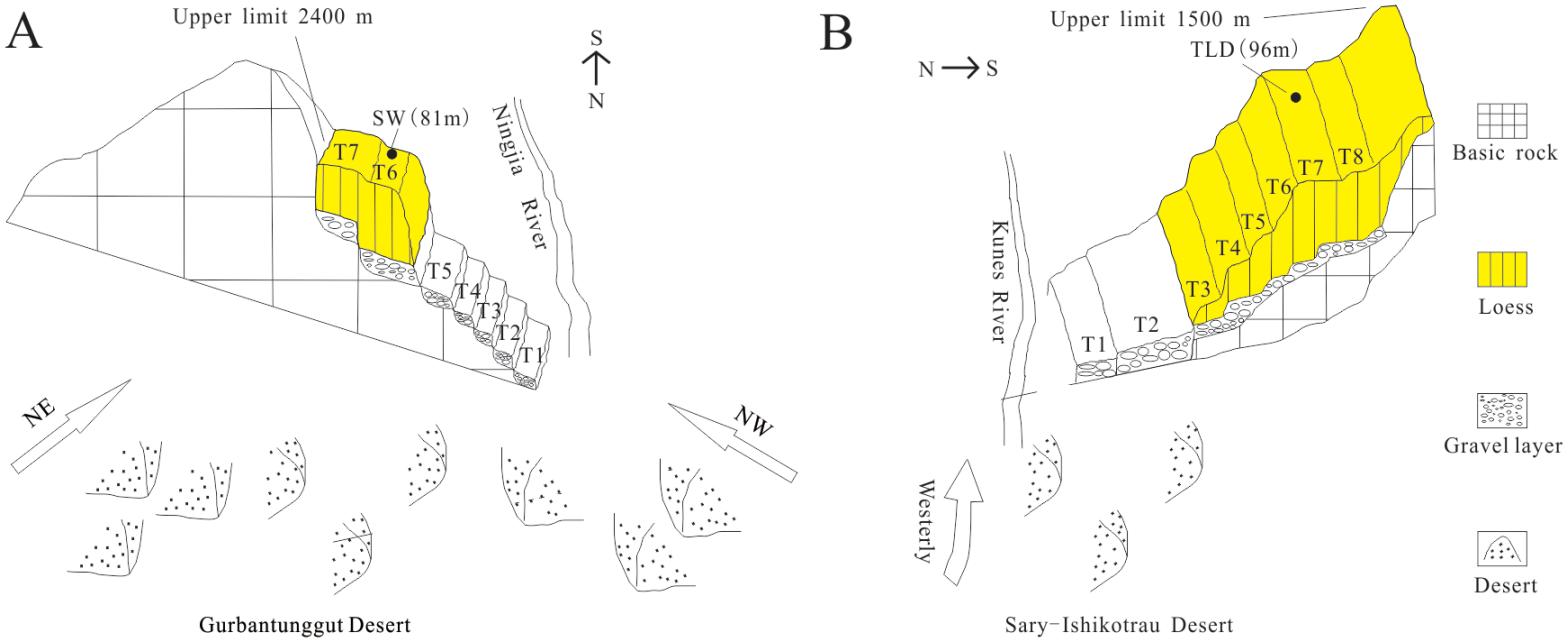
Two concept models showing the style of entrainment, transportation, and deposition of aeolian dust in the Junggar basin (A) and the Ili basin (B). TLD: Talede drilling; SW: Shawa drilling.

The contour lines in Fig 8A illustrate that there are two directions of dense contours around Shawan: northwest and NNE-SSW. These two directions are in line with the directions of regional dunes and chains [16], which indicates that the loess around Shawan was transported from the Gurbantunggut Desert by NW and NNE-SSW winds. Modern meteorological data [47] (Figs 2 and 3) show that North Xinjiang is mainly controlled by the north branch of the westerlies and the Siberian High Pressure, creating NW winds and NNE-SSW winds, respectively. These two currents (Fig 1) meet in the Shawan area, resulting in the thickest loess deposit (81 m) on the sixth terrace of the Ningjia River to the north of the Tianshan Mountains (Fig 9A). The distribution and thickness of three sections on the north side of the Tianshan Mountains (Fig 7) further support that the loess distributed on the terrace in the middle is the thickest, whereas near the Junggar Basin and the Tianshan Mountains, the loess becomes thinner. Taking the Qitai area into account, the >63 μm particle contours show a decrease from north to south, indicating that wind from approximately north dominates the Qitai area. However, due to the smaller area of desert to the north of Qitai (Fig 1), only less than 20 m thick loess has been deposited in this area. The spatial characteristics of the >63 μm grain-size contours around Tacheng resemble those of the eastern part of the Ili Basin with a closed-basin sedimentary environment. The regional topography around Tacheng (Fig 1) is similar to the Ili Basin, which looks like a trumpet, with the mouth facing towards the west. Thus, this area may mainly receive dust and water vapor derived from Central Asia and transported by westerlies. Unlike the 96 m in the eastern Ili Basin, the loess between Tacheng and Yumin is rarely more than 30 m thick, which could be the result of a smaller dust source to the west of the Tacheng area. No evident characteristics were observed around Bole because of fewer sampling sites (2 samples). Based on the topographic characteristics of the closed valley around Bole (Fig 1), the loess thins from east to west. Because the loess distribution around Bole is not located in a downwind direction of the Gurbantunggut Desert, we infer that the loess originates from fluvial sediment via local mountain-valley wind circulation, as evidenced by local meteorological data (provided by the China Meteorological Data Sharing Service System). This phenomenon is similar to that of the loess sediment in the Jinsha River Valley [69]. Moreover, Deng et al. [70] suggested the loess around Bole exhibited a nearby provenance based on rock magnetism and grain-size analyses.

A rapid decreasing trend in the >63 μm particle contours in the western Ili Basin suggests that the upwind Gobi deserts of Central Asia (e.g., the Sary-Ishikotrau Desert) are the primary dust sources of the Ili Basin via transportation by the westerlies [17, 19, 47] (Fig 9B). Because of the blocking effect of the Tianshan Mountains, abundant dust was deposited on the highest terrace of the Ili River around Qingshuihe, making it the thickest loess (202 m) deposit in the Ili Basin. In the eastern Ili Basin, along with increasing elevation, the >63 μm particle content decreases slowly and is approximately 20–10%. Fig 8C shows that the coarser particles appear to the west of Xinyuan rather than more proximally, and this figure also demonstrates that the loess around Xinyuan in the Ili Basin has a thickness of 96 m on the seventh terrace of the Kunes River (Fig 9B). Song et al [17] noted that, in general, the thickest loess is located around Xinyuan and thins to both sides. Due to their location in the lower reach of the Ili River, the few well-developed river terraces around Yining on the west of Xinyuan provide an adverse condition for dust accumulation, with the exception of the Qingshuihe loess hill (Fig 8A), which is closer to the source region. To the east of Xinyuan, due to the elevation increase, the energy of the westerlies decreases gradually, which means that only finer particles (Fig 8C) are carried to this high-elevation area. However, the Xinyuan zone is an ideal site for loess accumulation, due to the adequate wind energy and well-developed terrace of the Kunes River. Because of the high-elevation (1875 m) nature of the Zhaosu Basin, only several meters of loess cover the Tekes river terrace.

Between Hotian and Yutian, the contours of the >63 μm grain-size component are similar to those around Shawan, which can be correlated with northwest- and northeast-aligned dunes and chains (Fig 9A). This pattern indicates that this area is mainly dominated by two winds: the NW westerlies coming from the west over the Pamir pass and the NE winds passing through the Urumqi wind outlet in the Tianshan Mountains. These two winds meet in the Hotian-Yutian area and deposited the thickest loess sediment (670 m) on the flat terrace of the Keriya River. These winds even lift fine dust up to above 5000 m [15]. Based on 17 selected meteorological stations for the period from 1996 to 2000, Zhu et al. [71] suggested that the winds near the ground surface of South Xinjiang were mainly northeast and northwest wind systems due to the effects of the upper-air westerly circulation. However, between Kashi and Hotian and between the Yutian and Qiemo, the thickness of the loess gradually decreases and finally disappears because these locations are not downwind of the Taklimakan Desert.

Furthermore, we compared the Xinjiang loess with the well-studied Luochuan loess [72] in the CLP and the Tajik Karamaidan loess [73] in Central Asia (Fig 7). We found that although their thicknesses differ, their pedostratigraphies exhibit significant similar characteristics in the upper eight paleosol layers, and their defined B/M boundaries were located in loess L8. This similarity demonstrates that at the glacial-interglacial scale, the regional paleoclimates of not only the monsoon-dominated CLP but also westerlies-controlled Central Asia and monsoon/westerlies- dominated Xinjiang are basically controlled by global ice volume variations [74] or Northern Hemisphere summer insolation [75]. Fig 7 also shows that loess was deposited at least ~1 Ma in the Ili Basin, north of the Tianshan Mountains and Kunlun Mountains. This large-area loess-depositing event indicates that persistent aridification occurred because of the stepwise uplifts of Tibet [28], global cooling [76], and/or the appearance of the grassland and desert steppe environments [16]. However, the Kunlun loess started to be deposited in the beginning of the Pliocene, which suggests earlier aridification occurred in the Tarim Basin rather than in the Ili Basin and the Junggar Basin.

In summary, there are three foremost factors that determined the thickness, distribution and formation age of the Xinjiang loess [55], including: (i) a sustained source of dust, (ii) adequate wind energy to convey the dust and (iii) a suitable deposition site. However, we must note that our results lack certain sampling sites, especially in the Tarim Desert. We hope that we will investigate the grain-size spatial distribution of the Xinjiang topsoil in more detail in the future.

## Conclusions

The Xinjiang loess is mainly distributed on terraces of rivers, low uplands, piedmonts and the margins of deserts. The thickness varies from several to several hundred meters. The majority of the Xinjiang loess developed since the middle Pleistocene; however, the oldest Xinjiang loess was deposited during the early Pliocene. The deposition of the Xinjiang loess is attributed to persistent aridification and the appearance of the grassland and desert steppe environments. The variations in the >63 μm sand fraction in the grain-size contours can be used to infer the local major wind directions. It is also suggested that persistent drying, adequate wind energy and suitable accumulation conditions are the three main controlling factors that determined the distribution, thickness and formation age of the Xinjiang loess.
